# Hyperglycemia-Induced Aberrant Cell Proliferation; A Metabolic Challenge Mediated by Protein O-GlcNAc Modification

**DOI:** 10.3390/cells8090999

**Published:** 2019-08-28

**Authors:** Tamás Nagy, Viktória Fisi, Dorottya Frank, Emese Kátai, Zsófia Nagy, Attila Miseta

**Affiliations:** 1 Department of Laboratory Medicine, Medical School, University of Pécs, H-7624 Pécs, Hungary; 2 Department of Dentistry, Oral and Maxillofacial Surgery, Medical School, University of Pécs, H-7621 Pécs, Hungary

**Keywords:** diabetes, O-GlcNAc, proliferation, cell cycle, cancer, hyperglycemia

## Abstract

Chronic hyperglycemia has been associated with an increased prevalence of pathological conditions including cardiovascular disease, cancer, or various disorders of the immune system. In some cases, these associations may be traced back to a common underlying cause, but more often, hyperglycemia and the disturbance in metabolic balance directly facilitate pathological changes in the regular cellular functions. One such cellular function crucial for every living organism is cell cycle regulation/mitotic activity. Although metabolic challenges have long been recognized to influence cell proliferation, the direct impact of diabetes on cell cycle regulatory elements is a relatively uncharted territory. Among other “nutrient sensing” mechanisms, protein O-linked β-N-acetylglucosamine (O-GlcNAc) modification emerged in recent years as a major contributor to the deleterious effects of hyperglycemia. An increasing amount of evidence suggest that O-GlcNAc may significantly influence the cell cycle and cellular proliferation. In our present review, we summarize the current data available on the direct impact of metabolic changes caused by hyperglycemia in pathological conditions associated with cell cycle disorders. We also review published experimental evidence supporting the hypothesis that O-GlcNAc modification may be one of the missing links between metabolic regulation and cellular proliferation.

## 1. Introduction

For living organisms, one of the most basic survival skills is the ability to adjust their metabolism to the available resources. When molecules and chemicals required for energy and for the building blocks of cellular components are in excess, it is evolutionary advantageous to gather and use these molecules as fast and as effectively as possible. On the other hand, when resources are scarce, cellular metabolism needs to be reduced or diverted to alternative resources in order to survive. Consequently, a major determining factor in cellular proliferation is the amount of available nutrients. The first discovered and relatively simple gene regulatory mechanism is the Lac operon in prokaryotic cells that enables bacteria to switch to lactose metabolism in the absence of glucose [1,2]. Transition (transcription and expression of enzymes such as beta-galactosidase) to lactose metabolism is switched on only if it is a necessity since it requires time and energy, which will considerably slow down the proliferation rate of the bacteria [2,3].

Eukaryotic and mammalian cells possess much more complex systems to deal with either sub-optimal or surplus nutrients, and metabolism-fueled logarithmic growth is usually overruled by cellular differentiation and hormonal regulation. Nevertheless, nutrient-driven regulation is still an essential attribute of every living cell. Any disorders in either the nutrient sensing, in the signal interpretation/transduction, or in the adaptation of the metabolic state might lead to disturbances of the cell growth, proliferation or the mitotic activity.

The prevalence of metabolic disorders is on the rise. Most importantly, the prevalence of diabetes (especially type 2) is increasing around the world (from 4.7 to 8.5%) due to longer life-span, obesity, sedentary lifestyle, and unhealthy eating habits during the past decades [4,5,6]. Numerous studies showed that diabetes increases the risk of various diseases that are generally considered to be cell proliferative disorders [7,8,9,10]. Although the cause-and-effect relationship is not always clear or obvious, increased risk of neoplasia [11], impairment of tissue regeneration [12,13], and disturbed inflammatory responses [14,15,16,17,18] are known issues of diabetic complications. In this review, we list a number of proliferative disorders of which diabetes or insulin resistance is thought to play a role in the pathomechanism. We also present how the metabolic changes of diabetes and insulin resistance impact various intracellular regulatory pathways, nutrient sensing, and downstream signaling events. We discuss how the hyperglycemia-induced disturbance of these pathways, including protein O-linked β-N-acetylglucosamine (O-GlcNAc) regulation, may induce changes in the cell cycle regulation and cell proliferation.

## 2. Diabetes and Its Effect on Cell Cycle Regulation

According to WHO statistics, in 2014, 422 million people had diabetes worldwide, and the global prevalence among adults over 18 years of age was 8.5% [4]. Diabetes mellitus is a metabolic disorder characterized by chronic hyperglycemia, i.e. abnormally elevated level of glucose in the blood and interstitial fluids [19]. It is caused by decreased uptake of glucose by insulin sensitive cells (hepatocytes, myocytes, adipocytes) [20]. In type 1 diabetes, insulin production by pancreatic beta cells is deteriorated by a presumably autoimmune mechanism [21,22]. In type 2 diabetes, insulin resistance of the peripheral cells is the primary cause [23]. In the first phase, the body compensates insulin resistance by upping the insulin level until beta cells are exhausted and insulin drops below normal values [24]. Interestingly, recent development in neuropathological studies suggest that Alzheimer’s disease (AD) may have similarities with diabetes—hence, the proposed term for AD being type 3 diabetes [25]. AD is characterized by decreased glucose uptake of the neurons, similarly to the insulin-sensitive peripheral cells in type 2 diabetes. (However, it has to be noted that the extent of the involvement of insulin in the brain glucose metabolism is still far from completely understood) [26].

Despite the fact that the main problem of diabetes is that cells do not remove glucose from the blood efficiently, the deleterious effects and diabetic complications are due to elevated intracellular glucose (e.g., 2/3 of the advanced glycation end-products are produced intracellularly) [27]. This paradox can be explained by variances in insulin dependence between various cell types. While myocytes and adipocytes require insulin to facilitate glucose uptake, other cell types such as endothel cells and neurons do not need insulin for glucose uptake [25]. Not surprisingly, the most prominent pathological changes of diabetes are microangiopathies, neuropathies, and nephropathies [28]. In contrast, diabetic cardiomyopathy is rather caused by decreased glucose uptake (due to decreased GLUT4 membrane translocation upon insulin stimuli) and side-effects of the elevated insulin levels—decreased nitric oxide and increased intracellular Ca^2+^ [29].

Several pieces of direct experimental evidence suggest that hyperglycemia will lead to disturbed cell cycle regulation and proliferations even in non-malignant cells [30]. Altered cell cycle regulation has been observed in diabetic nephropathy [31], endothelial [9], and mesangial cells [32], as well as in astrocytes [33] and embryonic stem cells [34]. The contribution of hyperglycemia to enhanced tumor progression in malignant cells is also well known [8,35,36,37,38]. The underlying mechanisms are complex and deeply interconnected; evidence has been found that metabolic flux (increased substrate availability for nucleotide synthesis), oxidative stress, activation or inhibition of transcriptional factors, and various signaling cascades are all (at least partially) responsible for disturbing the cell cycle.

### 2.1. Metabolic Effects

Glucose, once it enters the cells, has a direct effect on cellular metabolism. It provides fuel for ATP generation, carbohydrate metabolites for protein post-translational modifications, glycolipids, and nucleic acids. Considering the latter, excess or the lack of glucose and its downstream metabolites could significantly influence DNA synthesis. Indeed, it was suggested a long time ago that manipulating glucose levels could differentiate malignant and non-malignant cells by measuring thymidine incorporation [39]. Another early study showed that brain tumor cells utilize more glucose (supposedly for increased nucleic acid synthesis) [40]. A more recent study found evidence that DNA synthesis and glycolysis is linked genetically and suggested that it is an adaptation mechanism in response to the energy provided by the environment [41]. Moreover, Ribose-5-phosphate, which is required for nucleotide synthesis and NADPH, is generated by the pentose phosphate pathway (PPP), the rate limiting enzyme being glucose-6-phosphate dehydrogenase. Takahashi et al. found that the flux through PPP greatly increased under hyperglycemic conditions in astroglia cells [42], although they assume that this effect is rather a transcriptionally regulated response (Nrf2 translocation to the nucleus) to oxidative stress than a metabolic effect. In another cell type (aortic smooth muscle cells), Peiro et al. also found that hyperglycemia is activating PPP by channeling the excess of glucose. They proposed that this mechanism may contribute to diabetic complications [43]. Through the progress of cell cycle, PPP is dynamically regulated, i.e., the highest flux through PPP occurs during the S phase [44] and contributes to cell cycle progression [45]. In malignant cells, PPP plays a part in the so-called Warburg effect and thus may contribute to tumor growth [46,47]. Warburg effect in essence is a switch from aerobic to anaerobic glucose metabolism even in the presence of oxygen (i.e., a less effective, “primitive” process) observed in malignant cells [48]. Interestingly, recent considerations suggest that normal cells can have a similar metabolic signature when entering proliferation (G_1_ phase) [49]. Some argue that rather than a consequence of malignant transformation, Warburg effect might preclude cancer [50]. Nevertheless, it seems to be that controlled metabolic change from aerobic to anaerobic glycolysis is also employed by normal cells for cell proliferative or other physiological purposes [51]. Taken together, it seems plausible that the excess of intracellular glucose is diverted to other metabolic pathways, such as PPP, and contribute to the initiation of the Warburg effect [47], which in turn have significant consequences on cell proliferation.

### 2.2. Oxidative Stress and Intracellular Consequences

Brownlee’s seminal work in *Nature* [27] proposed that oxidative stress and superoxide overproduction are the central elements of the diabetic complications. In short, excess intracellular glucose and increased flux through the tricarboxylic acid cycle overloads mitochondria with electron donors (NADH, FADH_2_) and increases membrane potential by accumulating protons in the intermembranous space. As a result, electron transfer is blocked at a certain threshold [52], and some of the electrons are used to generate O_2_- radicals. This free radical is then converted to H_2_O_2_ by superoxide dismutase (MnSOD). Eventually, H_2_O_2_ is converted by other enzymes to H_2_O and O_2_ [53]. Basically, the extra “fuel” of intracellular glucose is branching off at the electron transport system into reactive oxygen species (ROS) production instead of supplying further proton pumping. Interestingly, decreasing the membrane potential by ADP, Pi, or by transfection with uncoupling protein 1 (UCP-1) prevents ROS formation just as well as MnSOD overexpression does [54]. It seems to be that the proper function of ATP synthase is key in this process [55]. Decreased ATP synthesis has been found in diabetes [56] and in insulin resistance [57]. Since mitochondrial proton gradient also depends on ATP synthesis, its lower rate might contribute to increased ROS production [58]. On the other hand, enhancing the activity of ATP synthase by, e.g., exercise seems to have an inhibitory effect on oxidative stress [59,60].

An excess amount of H_2_O_2_ inside and leaking out of mitochondria introduces a number of detrimental effects; peroxidation of lipids, nucleic acids, and proteins might occur before free radicals are detoxified by glutathione peroxidase or catalase. Thus, increased ROS and oxidative stress are usually associated with cellular damage, apoptosis, or cell cycle arrest. However, ROS increases throughout G_1_, S, G_2_, and mitotic phases [61], where the mitochondria proliferation is also the highest [62]. One way that ROS influences cell cycle progression is by inactivating a protein complex called anaphase, promoting complex (APC) [61]. On the other hand, hyperglycemia-induced oxidative stress can also cause decreased proliferation [63], e.g. by increased expression of cell cycle inhibitor p21^cip1^ through the FOXO3A/ β-catenin signaling pathway [34]. These various effects of chronic hyperglycemia and associated oxidative stress on cellular proliferation might depend on the cell type, duration, and seriousness of hyperglycemia and/or ROS and the actual state of the free radical scavenge system [64]. Thus, severe damage to DNA due to toxic level of ROS will lead to apoptosis, while a moderate level of intracellular ROS might cause disturbances in the mitotic activity.

A key consequence of the overproduction of superoxide by mitochondria is its inhibitory effect on glyceraldehyde-3-phosphate dehydrogenase (GAPDH) [27]. This has a deep impact on the metabolic flux through glycolysis and its bypassing metabolic routes. The process is a self-stimulating mechanism because enhanced flux through these pathways also generates more ROS [65]. Since GAPDH is (partially) inhibited, glucose metabolites upstream of GAPDH are increased. For example, dihydroxyacetone phosphate (or glycerone phosphate) is an isomer of glyceraldehyde-3-phosphate, which is a substrate for various glycerolipid and glycerophospholipid synthesis. Diacylglycerol (DAG) is a direct activator of protein kinase C (PKC). Among the many targets of PKC, cyclins as well as cell cycle inhibitory proteins are also present. However, the cell cycle promoting or inhibiting effect of PKC is the sum of many factors, including the cell type and the PKC isoenzyme composition of the cell [66].

Methylglyoxal is another byproduct of glyceraldehyde-3-phosphate and dihydroxyacetone phosphate and is a toxic metabolite due to its capacity to react covalently with arginine, lysine and cysteine on proteins. These irreversibly modified proteins are called advanced glycation end-products (AGEs), and they may have altered or disabled functions compared to the non-modified form of the protein [67]. AGEs can induce oxidative damage as well [65,68]. Extracellular AGEs, which can form directly from glucose reacting with the proteins’ amino group through Schiff base and Amadori product, may also activate AGE receptors (RAGE) [69]. RAGE is a transmembrane receptor, and its activation leads to NF-κB nuclear translocation [70] and activation of Ras, which is a starting point of many signaling pathway, including the mitogen-activated protein kinase (MAPK), protein kinase C (PKC), phosphatidylinositol 3-kinase (PI3K), protein kinase B (Akt), and mammalian target of rapamycin (mTOR) [71]. Interestingly, RAGE signaling has been implicated in influencing both cell proliferation [72,73] and in cell cycle arrest [74].

In hyperglycemia, the excess amount of glucose is reduced to sorbitol by aldose reductase. Although sorbitol can be converted to fructose, which can be cycled back to glycolysis by hexokinase, the balance is tipped toward sorbitol in diabetes. Aldose reductase requires NADPH for its reaction. Some of the NADPH may be replaced by the PPP pathway, but the net effect will be an oxidative state due to the sorbitol overproduction and NADPH depletion of the polyol pathway. Sorbitol may also contribute to the oxidative stress by glycating proteins, producing AGEs, and activating RAGE signaling. A recent study revealed that the increased transcription of the polyol pathway gene aldo-keto-reductase-1-member-B1 (AKR1B1) promotes the epithelial-to-mesenchymal transition (EMT) in human lung and rodent colon cancer through autocrine TGFβ stimulation. According to these data, the polyol pathway serves as a molecular link between glucose metabolism and cancer differentiation and could be a novel therapeutic target [75]. Sorbitol is also often used in in vitro experiments to induce hyperosmotic stress [76]. In these experiments, p38 activation is regularly found to be mediating the effect of sorbitol. Apoptosis and cell cycle arrest are the mostly likely outcome [77], but the p38 pathway may have other effects on the cell cycle as well [78,79].

The fourth metabolic pathway involved in the by-passing of GAPDH and glycolysis inhibition is the hexosamine biosynthesis pathway (HBP). Normally, only a few percent of the hexoses is metabolized through this pathway, but a partial block after the branching off of the HBP will increase the substrate availability (fructose-6-P) for the first enzyme of the HBP: glutamine-fructose-6-phosphate amidotransferase (GFAT). The end-product of HBP is uridine diphosphate N-acetylglucosamine (UDP-GlcNAc), which is used in many post-translational modifications of proteins. While most of these modifications are permanent and concern membrane proteins and extracellular proteins, one particular type of glycosylation, called O-GlcNAc modification, modifies intracellular proteins and serves as a signaling mechanism [80,81]. So far, hundreds of proteins have been shown to be modified by O-GlcNAc, including transcriptional factors and several elements of signaling cascades [82]. O-GlcNAc seems to be a direct link between nutrient sensing and cellular metabolism. Increased O-GlcNAc modification due to elevated substrate availability influences insulin receptor signaling, protein degradation, cell proliferation, and mitotic activity. We demonstrate more details and evidence about O-GlcNAc modification’s influence on cell cycle regulation in the following sections.

### 2.3. Downstream Signaling Pathways Regulating Cell Cycle During Hyperglycemia

**Figure 1:**
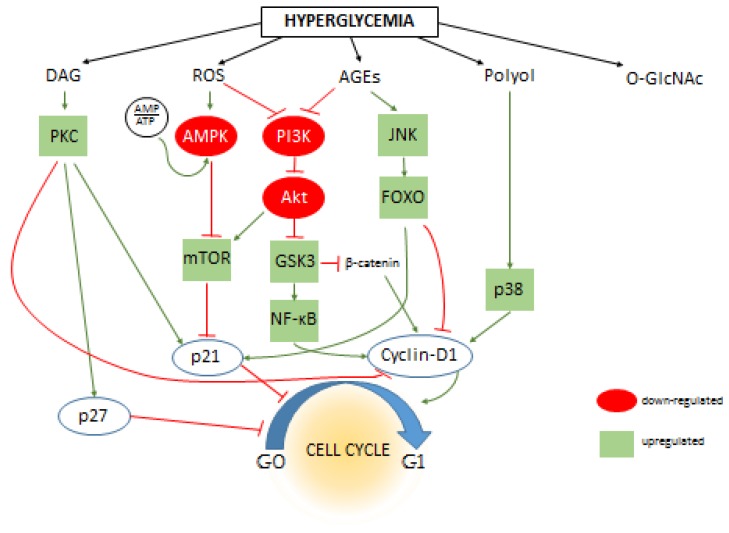
The effect of hyperglycemia on intracellular signaling pathways and cell cycle regulation. The primary metabolic changes caused by excess amount of intracellular are increased levels of diacyl-glycerol (DAG), reactive oxygen species (ROS), advanced end-glycation products (AGEs), sorbitol (Polyol), and protein O-Glycosylation (O-GlcNAc). Dozens of secondary messengers and signaling elements are activated (or de-activated) that are connected to the cell cycle regulatory system and eventually influencing cell proliferation by either directly influencing cyclins, cyclin-dependents kinases and cell cycle inhibitors such as p21 or altering their expression level through influencing transcriptional activity. The figure shows some of these connections; positive or negative effects inherent to the interactions are indicated by green or red lines between proteins, respectively. The effect of hyperglycemia (if it is known) on the activity of individual signaling elements are indicated by green (up-regulation) or red (down-regulation) background coloring. For clarity, the connections of O-GlcNAc are omitted from this figure, however please note that increasing number of evidences suggest that the majority of intracellular signaling elements are modified and influenced by O-GlcNAc modification.

The signaling pathways associated with the pathophysiological changes of hyperglycemia are numerous. Either by direct metabolic effect, by alternative metabolic pathways, or by oxidative stress, hyperglycemia may influence literally hundreds of various intracellular signaling routes, some of them converging to cause the same effects, some of them counteracting each other. In Figure 1, we summarize some of the signaling routes regulating cell cycle that could be influenced by the metabolic consequences of hyperglycemia. Most studies focus only on smaller pieces of the puzzle, thus clarifying the multi-layer cause and effect relationships (e.g. hyperglycemia initiates secondary metabolic changes such as ROS formation, which in turn impacts subsequent signaling pathways, leading to the direct influence of cell cycle regulating elements such as cyclins) would require more vertical, in depth analysis. Nevertheless, several studies identified secondary intracellular messengers that relay the effects of hyperglycemia to cell cycle regulation. For example, central elements of the insulin signaling pathway, PI3K and Akt, were implicated in several studies to conduct the effects of hyperglycemia on cell proliferation. Interestingly, some reported increased Akt activation and proliferation [30,83], while others showed the opposite; Akt was downregulated, and consequently, cyclin D was as well, while cell cycle progression inhibitor p27 was upregulated [9,84,85]. Along the PI3K/Akt axis, mTOR is an important intermediate element; it is a direct or indirect regulator of gene expression, glycolysis, mitochondrial activity, autophagy, or membrane transport processes. mTOR was also recognized as a nutrient sensing system. It can re-organize the distribution of resources and the cellular metabolism, but it also impacts cell proliferation and cell cycle regulation [86,87]. Inhibitors of mTOR are used in anti-cancer therapies and as immunosuppressants [88,89], and mTOR has been associated with the regulation of multiple elements of the cell cycle signaling including cyclins and p21 [90,91]. AMPK is another nutrient sensing mechanism (also interacts with mTOR). It is a kinase that is activated when the ratio of ATP/AMP decreases. Its downstream targets also include p53 and p21. The anti-diabetic drug metformin, frequently used in the therapy of insulin resistance and type 2 diabetes, seems to also act on AMPK, which eventually leads to the membrane translocation of the GLUT4 transporter. Interestingly, AMPK has been studied as a potential target for cancer therapy as well [92]. Moreover, studies found that metformin therapy decreased the prevalence of malignant diseases [93].

FOXO regulation is normally suppressed by Akt signaling, however in diabetes, Akt suppression could be weaker. Moreover, FOXO is activated by AGE and ROS formation through the JNK pathway. Activated FOXO is considered a cell cycle inhibitor by influencing several actors, such as upregulating p21 and cyclin-dependent kinase inhibitor while suppressing cyclin D1 and D2 [34,94]. A recent study by McClelland Descalzo et al. suggest that there is an interaction between FOXO and β-catenin [34]. According to their proposed mechanism, they form a complex and are translocated in the nuclei, their transcription activity leading to slower proliferation. Interestingly, β-catenin is usually implicated in enhancing cell proliferation as part of the Wnt pathway [95], but its effect on cell cycle regulation is probably much more complex than first assumed [96]. Since Wnt pathway can be inactivated by ROS formation, the β-catenin destruction complex is allowed to degrade β-catenin prematurely. However, Wnt activation by ROS has been also reported [97,98]. Glycogen synthase kinase 3 (GSK3) is part of the β-catenin destruction complex, which can prime β-catenin for degradation. Effects of hyperglycemia may also converge on GSK3 through the PI3K/Akt pathway. Although GSK3 is “constitutively” activated, under normal circumstances, it is also inhibited by Akt phosphorylation [99]. In diabetes, PI3K/Akt may be disrupted by ROS and AGEs [100,101]; thus, GSK3 is released from their inhibition. However, the regulation of PI3K/Akt by oxidative stress is more complex, and it may be stimulated by ROS just as well as inhibited [102,103]. As mentioned, activated GSK3 is regulating β-catenin, but several other targets of GSK3 are also known that are related to cell cycle regulation [104]. This wide range of functions of GSK3 may counteract each other, and the view seems to currently be shifting from considering it as a tumor suppressor [105] to a positive regulator of cell proliferation [106]. One possible explanation of GSK3 pro-oncogenic activity is that, despite its suppression on β-catenin and cyclin D, it also activates NF-κB [106], which promotes cell cycle progression at many targets [70].

## 3. Cell Proliferative Disorders Related to Diabetes

### 3.1. Cancer Development

Hyperglycemic conditions have been numerously reported to impact oncogenesis and tumor progression [30,107,108,109], and it was also observed that hyperglycemia can negatively influence the effect of anti-cancer therapies [110,111]. Tumor development, recurrence, metastasis, and fatal outcome have been shown higher occurrence in patients with diabetes or hyperglycemia [7,110]. Meta-analysis showed increased incidence of liver [112], pancreas [113,114], breast [115], kidney [116], colorectal cancers [117], and Non-Hodgkin’s lymphoma [118] among diabetic patients. Women with diabetic metabolic disorder have higher (20–28%) risk for breast cancer [115], which has been further linked to poor overall survival rate [119]. Hyperglycemia was also reported to change surface glycophenotypes both in vitro and in vivo to phenotypes typically present on malignant cells [120]. In A549 lung adenocarcinoma cells, increased amount of glucose uptake resulted in shunting glucose through HBP and in significantly higher amount of UDP-GlcNAc and its derivatives, leading to aberrant glycosylation [121]. Colon adenocarcinoma cells kept in high glucose and colon tumors from hyperglycemic mice presented more UDP-GlcNAc than those of cells cultured under low glucose and tumors of euglycemic mice [120]. Others also demonstrated that excess in UDP-GlcNAc and in its derivates due to hyperglycemia has an effect on changed protein glycosylation [122]. Many studies confirmed hyperglycemia-induced aberrant glycosylation is strongly associated with oncogenic transformation [123,124,125,126].

These findings indicate that there is a link between glucose metabolic disorders and tumor growth and behavior of cancer cells, but the complex molecular mechanisms remain to be mapped and analyzed. Selectively targeting pathways (metabolic or downstream signaling routes) will also require exploring the metabolic signaling pathways of individual malignancies. Nevertheless, there are some promising results with anti-diabetic drugs in the therapy of malignant disorders [127]. For example, the anti-hyperglycemic drug metformin was found to decrease the risk of diabetic patients with colorectal cancer [128]. Moreover, metformin administration seems to inhibit both cell proliferation in vitro and colon carcinoma growth in vivo [129,130]. Another antidiabetic drug called exenatide, which is “a glucagon-like peptide-1 receptor agonist, counteracts hepatocarcinogenesis through cAMP-PKA-EGFR-STAT3 axis” [131].

The effect of chronic hyperglycemia on the complex regulation of the immune system is beyond the scope of our present review, but excellent previous publications covered it in detail [132,133,134]. Evidence for an impact of glucose metabolism on the cellular proliferation of immune cells, e.g. neutrophils [135] or T-cells [136,137], has been also demonstrated. However, we would like to call attention to an interesting and important consequence of the altered immune regulation under diabetic condition; cancer development. It became evident in the last several decades that the immune system plays a crucial role in holding cancer development and progression at bay. In fact, the Nobel prize was awarded in 2018 for the “discovery of cancer therapy by inhibition of negative immune regulation” [138,139]. According to a recent report by Fainsod-Levi et al. [140], hyperglycemia might decrease neutrophil number and impair their mobilization, which could contribute to increased metastasis formation. Further studies will hopefully elaborate more on the issue of whether hyperglycemia-impaired immune functions are important contributors toward tumor development. Nevertheless, an increasing amount of evidence suggests that the immune system and inflammation is heavily influenced by metabolism, e.g. certain cell types known for augmenting anti-tumor immunity (e.g., T helper type 1) could be suppressed (or favored by specific therapy) based on their metabolic preference [141].

### 3.2. Skeletal Growth and Bone Remodeling

Diabetes has been associated with poor bone health. It has been proposed that diabetes affects bone growth, and children with juvenile diabetes have showed delayed skeletal maturation and altered craniofacial development [142,143,144,145]. Impaired osteoblast function due to insulin insufficiency increases the risk of low bone turnover, which eventually decreases pubertal bone mass and reduces bone mineral density during adulthood therefore predisposing to osteopenia and osteoporosis [142]. DeShields and Cunningham found lower bone mineral density at the femoral neck and total femur in diabetic US adults. Their results also indicated that osteoporosis is more likely (4.7 times higher risk) to occur in individuals suffering from type 1 than in those of with type 2 diabetes [146]. Weber et al., in a population-based cohort study by investigating the incidence of bone fractures in 30,394 type 1 diabetic participants and 30,3872 non-diabetic ones, also found that diabetes increases the risk of incident fractures. Furthermore, lower extremity fractures were more frequent in subjects with type 1 diabetes [147]. The proposed intracellular mechanisms by which hyperglycemia impacts osteoblasts and osteoclasts include many of the aforementioned cellular signaling elements such as mTOR, protein kinase B, or PI3K [145]. Among other effects (such as differentiation toward adipocytes instead of osteoblasts), diabetes may also disturb osteoblastogenesis by influencing FOXO regulation and subsequently cell proliferation [148,149].

Similar to skeletal bone health, diabetes has also been linked to oral diseases, including periodontitis, which is characterized by alveolar bone resorption and supporting tissue destruction around the teeth [150]. Since 1993, periodontitis has been referred as the “sixth complication of diabetes” [151]. Poor glycemic control in type 2 diabetes have been found to be positively associated with increased alveolar bone loss and more severe progression of bone resorption [152]. Furthermore, studies have suggested that diabetic condition not only induces periodontal problems but can also alter orthodontic tooth movement within the alveolar bone [153,154,155]. During orthodontic therapy, force generates a complex adaptive response in the surrounding tissues and bone which is disturbed in diabetics. Furthermore, by reversing the diabetic state, both insulin alone and adjuvant metformin as an add-on to insulin therapy could improve the response of periodontal structures when exposed to orthodontic forces [154,155]. Included in the proposed mechanisms responsible for the deleterious effects of diabetes on periodontal conditions is the disturbance of the proliferation capacity of fibroblasts specific for periodontal ligament, as the results of several studies suggest [156,157,158,159].

### 3.3. Insufficient Tissue Regeneration

Tissue regeneration requires well-coordinated interactions of various cell types, including fibroblasts, endothelial cells, cells of the immune system, and the damaged tissue itself. Diabetic patients are at higher risk for developing non-healing wounds and skin ulcerations. For example, a diabetic foot ulcer is a major complication, and approximately 15–25% of diabetics are affected [160,161]. Several studies have highlighted the combined importance of poor healing capacity and infection susceptibility. However, the mechanisms underlying this phenomenon are not fully understood. Diabetic complications such as neuropathy, vascular damage, and an increased systemic inflammatory state certainly contributes to the development of diabetic foot ulcers [162]. The role of the local effects of hyperglycemia has to be considered as well (ROS, AGE formation). At the cellular level, a main contributor of impaired wound healing and dysfunctional epithelization is the altered epigenetic regulation, which ultimately can impede keratinocyte, fibroblast, and macrophage function [163]. In wound healing processes (matrix deposition, remodeling), fibroblasts play a key role, so any impediments to their function will result in non-healing wounds, insufficient tissue repair and regeneration. Studies have confirmed that high glucose concentration initiates a complex downstream cascade of molecular disturbances of the dermal fibroblasts [164]. Fibroblasts exposed to high glucose become reactive but not active [12], disrupting the normal cell physiology. Hehenberger and coworkers found that high glucose concentration inhibited fibroblast proliferation and that cells became resistant to proliferate when exposed to certain growth factors (IGF-1, EGF) [165]. Goldstein et al. also confirmed decreased growth capacity of diabetic cutaneous fibroblasts [166]. Investigators also confirmed reduced proliferative, collagen synthetic, and secreting capabilities of diabetic skin fibroblasts [167,168]. Interestingly, others found contradicting results, i.e. impaired cellular migration in dermal fibroblasts harvested from diabetic mice was more prominent while the disturbance in cell proliferation was not a significant contributor [169].

Hyperglycemia has been shown to affect neovascularization by causing abnormal endothelial cell function (recruitment, proliferation, migration, collar stabilization, and cooping) [170]. Both micro- and macrovascular complications have been confirmed to be related to poor glycemic control in patients with both type 1 and type 2 diabetes [170]. Studies have shown that endothelial progenitor cells (EPCs), which are the main source of endothelial cell repair and important regulators of angiogenesis, are significantly decreased in bone marrow and/or dysfunctional under diabetic conditions [171,172,173]. Loomans et al. found a reduced number (44% less) of EPCs obtained from patients with type 1 diabetes when compared to control subjects. Furthermore, as confirmed by in vitro angiogenesis assay, their function was also impaired [172]. Cell proliferation and differentiation is mainly restricted due to excessive hyperglycemia-induced ROS production, which results in p38 MAPK activation [174]. Via MAPK pathways, AGEs can increase EPC apoptosis and decrease nitric oxide release. Shen et al. cultured EPCs with various concentrations of AGEs in the presence or absence of MAPK (ERK/p38/JNK) inhibitors and found that AGE treatment disrupted EPCs cell physiology, which was accompanied by a downregulation of eNOS and Bcl-2 expressions as well as an elevation in Cyclooxigenase-2, Bax, NF-κB, and Caspase-3 in a MAPK-dependent manner [174]. Varma et al. studied the PI3k and Akt signaling pathway and found that hyperglycemia decreased Akt activity and proliferation in human umbilical vein endothelial cells (HUVEC) [9]. Others also demonstrated that high glucose concentration affects endothelial cell cycle, increase DNA damage, and induces cell death [175]. Furthermore, hyperglycemia decreases dermal microvascular endothelial cell proliferation by 39% and tube formation 42% when compared to normoglycemia [176].

### 3.4. Renal Mesangial Cell Growth

Diabetes and hyperglycemia are also two important factors playing a role in the development of chronic kidney disease [177]. Diabetic nephropathy (DN) has been reported to increase mortality rate, as it has become the major contributing cause of end-stage renal disease [178]. About 30% of the patients suffering from diabetes for 20 years will develop DN [179]. Glomerular matrix protein such as fibronectin accumulation and alteration in mesangial cell proliferation and hypertrophy are the major contributors of DN [180,181]. In his study, Wolf showed that mesangial cells are arrested in the G_1_ phase after some active self-limited proliferation and undergo hypertrophy due to an increase in the synthesis and deposition of fibronectin [31]. According to this, the increase of mesangial cell mass is a result of a limited and transient proliferation, followed by growth arrest and hypertrophy [31,179]. In some studies increased glucose levels expressed antiproliferative effects [179,182,183], but in other studies, it rather induced mesangial cell proliferation [180,184,185]. Li et al. even found that betain, a zwitterionic quaternary ammonium salt compound, decreased the proliferation and induced G_1_-phase arrest significantly in mesangial cells cultured under high glucose condition [184], indicating that betaine can be protective against high glucose–induced cell proliferation and extracellular matrix accumulation.

## 4. O-GlcNAc and Its Effect on Cell Cycle Regulation

O-GlcNAc modification is a dynamic post-translational modification (PTM) affecting serine (Ser) and threonine (Thr) amino acids of nuclear, cytoplasmic, and mitochondrial proteins. The attachment and removal of the O-linked β-N-acetylglucosamine moiety is performed by only one pair of enzymes, the O-GlcNAc Transferase (OGT) and O-GlcNAcase (OGA), respectively. Its targets include a wide variety of proteins involved in the regulation of gene expression, protein translation, degradation, signal transduction, cell cycle regulation, etc. [186].

**Figure 2:**
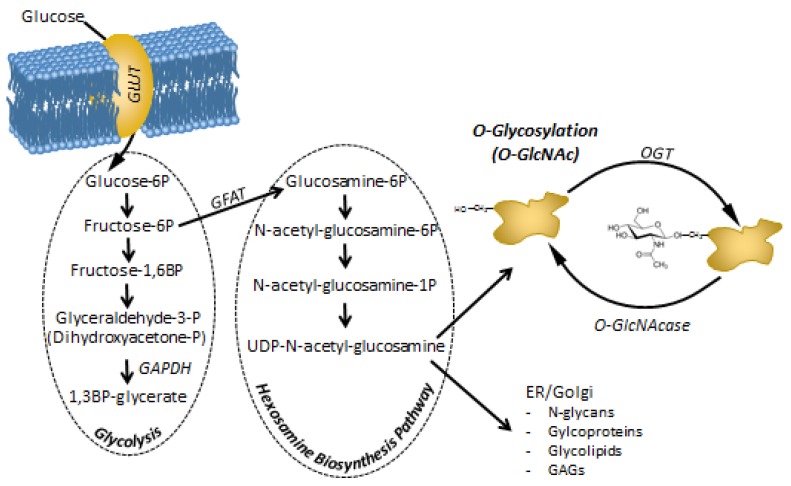
Metabolites of the hexosamine biosynthesis pathway (HBP) and protein O-Glycosylation (O-GlcNAc) modification. HBP branches off from glycolysis at fructose-6P. Thus, the amount of the end-product of HBP; UDP-N-acetyl-glucosamine (UDP-GlcNAc) depends on the rate of glucose entering the cells, but also on the rate of glycolysis that process the majority of fructose-6P. Increased glucose uptake or a block in glycolysis (e.g. inhibition of GAPDH by ROS) will increase the flux through HBP. It has to be noted that lipid (acetylation), protein (transfer of the amino group from glutamine) and nucleotide (linkage of N-acetyl-glucosamine to UDP) homeostasis may also influence HBP apart from carbohydrate metabolism [189]. UDP-GlcNAc is a substrate for many complex biomolecules and post-translational modifications. In particular, protein O-GlcNAc. As O-GlcNAc is recognized to modify and influence hundreds if not thousands of proteins, piling evidence suggests that it may be a direct mediator and feed-back mechanism between metabolic challenges and cellular adaptation and regulatory functions, including cell proliferation.

As the modification’s donor substrate, UDP-GlcNAc represents the end-product of the HBP, integrating nucleotide, glucose, amino acid, and free fatty acid metabolism. O-GlcNAc modification is highly responsive to the cell’s metabolic state and nutrient availability (Figure 2). Since approximately 2–5% of glucose influx is directed toward this pathway [187], hyperglycemic conditions (e.g., in diabetes mellitus) result in increased HBP flux together with consequently elevated O-GlcNAc levels. Additionally, regarding the excessive glucose influx, the augmented superoxide production and oxidative stress in chronic hyperglycemia also trigger O-GlcNAc modification and HBP by the inhibition of GAPDH [53]. Blocked GAPDH function next results in a high level of fructose-6-phosphate production, providing more substrate for the rate-limiting enzyme of the HBP; namely, GFAT. Moreover, high oxidative stress also strengthens the inhibitory interaction of fatty acid synthase with OGA, correspondingly resulting in elevated O-GlcNAc [188].

Elevated HBP flux and O-GlcNAc modification in turn has been shown to contribute to the development of insulin resistance in adipocytes and myocytes [187,190]. Several studies have shown that the elevation of HBP flux in different ways (through excessive amount of glucosamine, free fatty acids, or palmitate) results in a reduced insulin sensitivity in these tissues [190,191,192], while the overexpression of GFAT or OGT leads to the same consequences [193,194].

During the cell cycle coordination, swift responses to the changing environment or cellular damages are essential. Thus, post-translational modifications that allow dynamic, often reversible regulation have a crucial role in this complex process. The importance of O-glycosylation in cell division became evident in the last few years [195]. It has been shown that the OGT, OGA, and O-GlcNAc levels significantly fluctuate during the cell cycle, thereby nutrient availability—mediated by O-GlcNAc modification—may influence the progression of cell division [196,197,198,199]. Accordingly, in all human cancer types studied so far, a disturbed O-GlcNAc cycling or altered expression of O-GlcNAc enzymes has been shown [200].

Numerous cell cycle regulators have been identified as O-GlcNAc substrates. Among the key regulatory elements, cyclin D1 has been shown to be influenced by O-GlcNAc. Out of the three types of D cyclins controlling the G_1_/S transition in mammals, Cyclin D1 is the most frequently dysregulated, and its abnormal function has been extensively studied in human malignancies [201]. Experiments using serum stimulation, a common method to trigger cell cycle entry, have shown a significant overexpression of OGT. Conversely, OGT blockade caused by either serum starvation or siOGT resulted in inhibited cyclin D1 expression together with reduced activity of PI3K, a well-known cyclin D1 regulator, demonstrating the link between the nutrient sensor modification and cell cycle progression [198,202]. Furthermore, β-catenin, a major cell cycle factor whose one key target is cyclin D1, is also influenced by OGT. Namely, O-GlcNAc modification of the protein negatively regulates its nuclear level. Normal prostate cells have been shown to have remarkably higher levels of O-GlcNAcylated β-catenin together with its slight nuclear localization compared to prostate cancer cells [203].

The expression of cyclin-dependent kinase 4 (CDK4), whose activity is regulated by Cyclin D, is itself also influenced by O-GlcNAc. A higher expression of both cyclin D1 and CDK4 was found in mouse embryonic stem cells after glucosamine treatment, together with the elevated expression of other contributors of G_1_/S transition control, CDK2, and cyclin E [204]. The beginning and progression of mitosis is highly dependent on the maturation promoting factor consisting of cyclin B1 and CDK1, two O-GlcNAc-influenced proteins. Cyclin B1 expression decreases in the case of either OGT or OGA inhibition, revealing the importance of both enzymes in the process [205]. Overproduction of OGT also led to the diminished expression of Cell Division Cycle-25 (CDC25), a phosphatase protein activating the CDK1/Cyclin B complex [206].

Because it belongs to another essential group of cell cycle regulating kinases (the Polo-Like Kinase (PLK) family), the PLK1 protein has a crucial role in the metaphase to anaphase transition. It phosphorylates and thus activates CDC25B (a CDC25 isoform), and its overexpression promotes cancer development [207,208]. The mRNA and protein levels of PLK1 were reported to be diminished after OGT overproduction. Besides, the G_2_/M-specific expression of PLK1 depends on p53 being itself an O-GlcNAc target [209]. Overall, this complex regulatory cascade is influenced by O-GlcNAc modification at various levels.

The third prominent mitotic kinase, the microtubule-associated Aurora B, has been revealed to be in complex with OGT, OGA, and protein phosphatase 1 (PP1) at the midbody during mitosis. The complex consisting of the four enzymes has been assumed to regulate the phosphorylation and O-GlcNAc modification of vimentin and the stability of the midbody during mitosis. In concordance, OGT overexpression perturbs cytokinesis and promotes aberrant chromosome number [206]. Similarly, OGA knockdown also promoted spindle defects [210]. Interestingly, Aurora B inhibition led to abundant OGT and consequent O-GlcNAc levels as well as hindered localization of OGT to the mitotic spindle, raising the question if OGT or OGA itself may be phosphorylated by Aurora B [211]. At the same time, OGT overproduction lowers Aurora B expression [206]. Further evidences also suggest that the construction of the mitotic spindle, the microtubule attachment to kinetochores, and chromosome alignment orchestrated by Aurora B and other mitotic kinases are under strict O-GlcNAc regulation [212]. At the other end of the mitotic spindle, the expression of centrosome localized Nuclear Mitotic Apparatus Protein (NuMA) was found at an increased expression level in OGA knockdown cells [210]. Magescas et al. demonstrated that spindle pole cohesion requires the association of NuMA and Galectin-3, but the loss of an O-GlcNAc modification site on NuMA (S1844A) disrupts this association and results in a phenotype similar to NuMA depleted cells (multipolar cells) [213].

O-GlcNAc modification regulates chromatin dynamics through its effects on the mitotic spindle and also affects histone proteins. Modifications at Lys-9, Ser-10, Arg-17, and Lys-27 of the most extensively studied histone H3 have been proven to be influenced by OGT [214]. O-GlcNAc sites on histones H2A, H2B, and H4 have also been mapped by mass spectrometry. Global histone acetylation is reduced after OGT overexpression [199]. Another family of chromatin-binding proteins is the MiniChromosome Maintenance (MCM) group. Several subunits of the MCM2–7 complex, which is crucial for DNA replication, are O-GlcNAc-modified. It has been reported that a stable interaction exists between OGT and distinct MCM subunits and that, after OGT silencing, chromatin-binding of MCM2, MCM6, and MCM7 are decreased [215]. During the anaphase of the mitosis, O-GlcNAc has a pivotal role in the regulation of the anaphase promoting complex/cyclosome (APC/C) activity via the modification of its co-activator, Cdc20 homologue 1 (CDH1). APC/C, together with its co-activators (Cdc20 and CDH1), orchestrates mitosis through ubiquitination of specific proteins, thus designating them for proteasome degradation. The O-GlcNAc modification of CDH1 antagonizes its phosphorylation and promotes APC/C-Cdh1 activity [216].

Apart from the numerous specific cell cycle regulators, several transcription factors involved in cell proliferation control are O-GlcNAc modified, e.g. p53, c-Myc, NF-κB, FoxM1, β-catenin, and Sp1 [205,217,218], as well as the tumor-suppressor retinoblastoma (Rb) [219] and cell cycle inhibitor p27 [220]. p21 protein was not demonstrated yet to be directly influenced by O-GlcNAc, however OGT knock-down was shown to up-regulate p21 while simultaneously down-regulate Cyclin D1 [221].

## 5. Cell Proliferative Disorders as a Consequence of O-GlcNAc Disturbances

Aberrant O-GlcNAc modification has been implicated in the etiology of human diseases, including diabetes, cancer, aging, cardiovascular disease, and neurodegenerative disease [222,223,224,225]. In these pathological processes, the metabolic dysregulation is increasingly recognized as a major component. An increasing amount of evidence shows that O-GlcNAc plays an essential role in mediating the effects of metabolic events to intracellular regulatory processes.

### 5.1. Pancreatic Beta Cell Regulation

O-GlcNAc plays an important role in the regulation of pancreatic beta cells; the enzymes OGT and O-GlcNAcase are in the highest amount relative to other cell types [226]. Hyperglycemia elevates O-GlcNAc levels in pancreatic beta cells, which seems to modulate insulin production and release, but the exact site(s) of interference has not been clarified yet; among others, modifying transcriptional factors and epigenetic regulations have been proposed [227,228]. Glucose levels and responsive O-GlcNAc modification influences insulin secretion and also has an important and specific role in beta cell development by activating the transition of positive endocrine progenitors into beta cell development [229]. Experimental data showed that beta cell specific transcription factors, Pdx-1, MafA, and NeuroD take part both in insulin gene transcription and in beta cell function by glucose regulation. Changes in glucose concentration can cause nuclear transport of NeuroD1 via its O GlcNAc modification. This protein is a direct target of Neurogenin 3, which is a main marker for monitoring pancreatic endocrine cell differentiation [230]. The expression of MaFA, which is essential in beta cell survival and insulin gene transcription [231], is also glucose dependent and requires O-GlcNAc modification, but the mechanism is unknown. Pdx-1, an insulin promoter factor necessary for beta cell maturation, is also modified by O-GlcNAc modification [227]. Interestingly, long-term elevation of O-GlcNAc induces beta cells death by apoptosis [232].

### 5.2. Cancer Development

Elevated glucose or glutamine levels are necessary for tumor cells to maintain their energy, carbon, and nitrogen generation pathways, which enable the rapid growth and proliferation of tumor cells. The direct substrate of O-GlcNAc modification, UDP-GlcNAc is in the focal point of many metabolic pathway, as carbohydrates, amino acids, lipids, and nucleotide metabolisms all affect its synthesis [80,233]. Not surprisingly, there is a growing interest to study metabolism fueled O-GlcNAc modification in malignant cells [224]. Revealing the behavior of O-GlcNAc in malignant tissues would help to design alternative therapeutic strategies but also improve diagnostic and prognostic tools. Many studies have revealed an increased level of OGT or protein O-GlcNAc in high-grade breast, colon, and prostate cancer when compared to low-grade ones [234]. Elevated O-GlcNAc has been described in other various cancers as well [235]. Tumor cells can elevate the total O-GlcNAc levels by increasing OGT or decreasing OGA, but for some tumors, O-GlcNAc deregulation is also known. Increasing O-GlcNAc and OGT may be also involved in tumor invasion and metastasis. Table 1 lists some of the recently published data about O-GlcNAc involvement in various types of neoplasia. As the data shows, there is a lot of discrepancies and contradiction between various experiments concerning the functional effects of O-GlcNAc (which could be certainly attributed to high level of variation among cancer types), but the majority of the studies demonstrated that overall O-GlcNAc levels are increased in tumor cells.

### 5.3. Embryonal Development

OGT and OGA are abundantly expressed in placenta, and O-GlcNAc modification apparently plays a significant role in placental function and placental development [248,249]. It was shown in mice models that OGT gene was also essential for embryonic stem cell viability [250], and the deletion of OGA has also proved to be fatal [251]. In oocytes, the regulatory role of O-GlcNAc during meiosis was recognized early, as disruption of O-GlcNAc interactions lead to delayed maturation [252]. This was further supported by additional studies [253,254], and a recent report also demonstrated that altered O-GlcNAc levels during oocyte maturation may negatively impact fertilization [255].

O-GlcNAc can affect several stages of pregnancy. In the pre-implantation phase, hyperglycemia have a negative effect on embryos [256]. Pantaleon et al. demonstrated in mouse zygote cultures that dysregulation of HBP and O-GlcNAc is a major contributor to the embryotoxic effects of hyperglycemia in early stage of pregnancy [257]. O-GlcNAc also plays an important role in pluripotent embryonic cell differentiation. Andres et al. showed that inhibiting OGT prior to neuronal induction of human embryonic stem cells caused an immature state of the cells [258,259]. O-GlcNAc is also influential for the neuronal differentiation at a later stage. Kim et al. demonstrated that in vitro neural stem cells under hyperglycemic conditions presented a global O-GlcNAc increase by enhanced OGT activity, which resulted in neural tube defects. They also proved that inhibition of OGT might be beneficial to protect from birth defects in diabetic pregnancies [260]. Another study also concluded that high O-GlcNAc levels due to diabetes might be the underlying cause for various neurodevelopmental disorders. [261]. Taken together, dysregulation of O-GlcNAc seems to be a very important regulatory mechanism in metabolically compromised pregnancies, contributing to the occurrence of various birth defects, including neuronal impairments.

### 5.4. Endothelial Cell Proliferation, Wound Healing

In neoplastic tissues, altered O-GlcNAc modification could not be a significant contributor of cancer cell proliferation, as we discussed above. However, angiogenesis, and vascularization is also required for “successful” tumor progression, invasion and metastasis. Lynch et al. studied the role of OGT in the growth, invasion and angiogenesis of human prostate cancer via regulation of FoxM1 and its downstream effectors. FoxM1 is an oncogenic transcription factor of invasion and angiogenesis. They found that reducing O-GlcNAc by shRNA inhibition of OGT in prostate cancer cells led to increased FoxM1 protein degradation. More importantly, OGT inhibition in prostate cancer cells decreased angiogenesis when overlaid on HUVEC cells [262]. Interestingly, Zibrova et al. found opposite results when O-GlcNAc was inhibited directly in HUVEC cells. They found that inhibition of GFAT, the rate limiting enzyme of HBP decreased O-GlcNAc but improved angiogenesis, and they reasoned that activation of AMPK targets GFAT for phosphorylation may lead to decreased O-GlcNAc levels in endothelial cells [263]. Earlier, the findings of Lou et al. also support the theory that increased O-GlcNAc impairs angiogenesis [264]. According to their data, both in cultured HUVEC and EA.hy926 endothelial cells and in STZ induced diabetic mouse aortic rings, glucosamine, and/or high glucose levels increased protein O-GlcNAc modification but inhibited cell migration/wound closure and capillary-like structure formation. They have also found evidence that O-GlcNAc’s effect is at least mediated via the Akt signaling pathway.

Increased O-GlcNAc has been also implicated in impaired wound healing [265]. It is well known that hyperglycemia alone or in combination with venous or arterial diseases, infection, and metabolic disease may cause delayed healing or non-healing chronic wounds [266]. Although O-GlcNAc’s impact on cell migration seems to be prominent in wound healing, its other effects on cellular adhesion or cell proliferation cannot be excluded [267]. Wound healing is a complex process that involves the mobilization and proliferation of keratinocytes, platelets, macrophages, endothelial cells, and fibroblasts [268]. Given that O-GlcNAc is a ubiquitous modification present and dynamically changing in all of these cell types, it is quite likely that its influence will be found in multiple processes of wound healing.

### 5.5. Immune Cell Proliferation

The presence of protein O-GlcNAc modification was reported from several cell types of the innate and adaptive immune system and also reviewed in recent publications [269,270]. Swamy et al. [137] characterized the O-GlcNAc regulatory enzyme OGT as it “acts as a master regulator that depends on nutrient levels to control the commitment of T cells to metabolically demanding processes of clonal expansion, self-renewal and differentiation”. A recent paper by Machacek et al. [271] proposed O-GlcNAc as a missing link between overnutrition and T cell function, i.e. they found that O-GlcNAc elevation favored the activation of pro-inflammatory Th17 cells. Although this was mostly attributed to IL-17 overproduction, a small increase in CD4^+^IL-17^+^ cells was also observed. In the case of B cells, the presence of OGT is required for B-cell homeostasis and activation, as reported by Wu et al. [272]. So far, O-GlcNAc has been demonstrated to have both anti-inflammatory and pro-inflammatory potential, depending the cell type and environmental factors. To make things even more complicated, both pro- and anti-inflammatory effects has been found to be mediated by NF-κB transcriptional activity [269]. Nevertheless, cellular proliferation is one of the key functions of immune cells that could be negatively (or positively) influenced by O-GlcNAc, whether it occurs during maturation, differentiation or activation and expansion [273,274].

The detection and measurement of O-GlcNAc levels in leukocytes has been suggested for the diagnosis of diabetes and pre-diabetic conditions [275]. Although elevated levels of O-GlcNAc in leukocytes in diabetic conditions were confirmed by others as well [276], its impact on the immune system are largely unknown. Similarly, compromised O-GlcNAc regulation in malignant disorders was also recognized and started to attract more scientific interest recently, including hematopoietic malignancies. E.g. high levels of O-GlcNAc was found in pre-B acute lymphocytic leukemia, which was associated with overactivation of the PI3K/Akt/c-Myc pathway and enhanced proliferation [277]. Elevated level of O-GlcNAc and/or increased expression of OGT transcripts was also found in acute myeloid leukemia (AML) [246] and in chronic lymphocytic leukemia (CLL) [245].

## 6. Conclusions

According to the frequently repeated phrase, “you are what you eat”. Although laymen often interpret this expression in a literal sense, the impact of nutrition on health cannot be denied. The lack or excess of one or more nutritional sources will indeed have a significant impact on the physiology of cells. In diseases of metabolic origin such as diabetes, metabolic changes may have direct harmful impacts on cells e.g. osmotic stress, energy depletion, toxic degradation products, etc. However, the alterations caused in normal cellular metabolic routes and regulatory pathways (which were evolved in fact to overcome environmental challenges, including metabolic ones) are just as important in the understanding of the pathophysiological mechanisms.

As we have summarized above, these very complex and interconnected pathways could and indeed do impact cellular proliferation, cell cycle regulation. Diabetes has been found to increase cancer incidence [278], renal hypertrophy [31] or vascular cell proliferation [279]. Whether the effect is pro- or anti-proliferative depends on the cell type, the duration of the changes, the extent of “damage” and probably numerous additional factors as well. Thus, although advancement in proteomics and metabolomics did help us to clarify and map many of these interactions, exponentially more data are still required to resolve contradictory results and clarify which pathway(s) are dominant under various conditions and how these pathways shift their influence as circumstances change. Focus on research of protein O-GlcNAc modification will probably be an increasingly important subject in this respect. Its ability to modulate almost all protein that can be phosphorylated (and probably even more as every Ser/Thr amino acids are potential targets for O-GlcNAc modification) and its direct dependence on carbohydrate substrate availability makes it an ideal candidate for making the link between nutrition and proliferation. Indeed, there is a growing number of studies demonstrating that metabolic changes in malignant cells come along with altered O-GlcNAc regulation [237,277,280,281]. Assessing O-GlcNAc levels in tissue samples via biopsies would help to grade the tumor and to give a better prognosis. Moreover, specific inhibitors of OGT and OGA are currently under development [282,283,284], offering promising future options for engaging metabolic regulation of cancer cell during cancer therapy.

## Figures and Tables

**Table 1:** Altered O-GlcNAc levels found in various types of neoplasia.

Cancer Type	Change in O-GlcNAc	Proposed Effects	References
Colorectal cc.	increase	Increased cell migration by up-regulating of β-catenin and E-cadherin levels.	[236]
Ovarian cc.	decrease	Loss of stability and nuclear translocation of tumor suppressor p53.	[237]
Prostate cc.	increase	Increased cell migration by down-regulating E-cadherin levels (contradicting data found in [236]).	[238]
Prostate cc.	increase	Promotes Bmi-1 stability and its oncogenic activity.	[239]
Pancreatic cc.	increase	Increased oncogenic NF-κB transcriptional activity. O-GlcNAc modified FOXO3 suppresses p21 thus cell cycle is accelerated. Stabilization of oncogenic transcription factor Sox2 by O-GlcNAc modification.	[240] [241] [242]
Breast cc.	increase	Tamoxifen resistance by reducing expression level of estrogen receptor alpha.	[243]
Lung and colon cc.	increase	Increased invasion and enhanced anchorage-independent growth	[244]
CLL	increase	p53, c-myc and Akt were O-GlcNAc modification. O-GlcNAc levels did not correlate with the clinical aggressiveness of CLL.	[245]
AML	increase	Increased cell proliferation and sustained undifferentiated state.	[246]
Lung metastasis of cervical cc.	increase	O-GlcNAc modification of NF-κB upregulates CXCR4 chemokine receptor.	[247]

The types of cancer (tissue samples or cell lines of oncogenic origin), the direction of change in protein O-GlcNAc modification (increase or decrease) and specific targets of O-GlcNAc modification (if available) are listed.
